# Concomitant Tickborne Encephalitis and Human Granulocytic Ehrlichiosis

**DOI:** 10.3201/eid1103.040776

**Published:** 2005-03

**Authors:** Stanka Lotric-Furlan, Miroslav Petrovec, Tatjana Avsic-Zupanc, Franc Strle

**Affiliations:** * University Medical Centre Ljubljana, Slovenia;; † Medical Faculty of Ljubljana, Ljubljana, Slovenia

**Keywords:** Anaplasma phagocytophilum, human granulocytic ehrlichiosis, Tickborne encephalitis, Slovenia, concomitant infection

## Abstract

We report a patient with febrile illness and epidemiologic and clinical findings consistent with human granulocytic ehrlichiosis and tickborne encephalitis, in whom infection with *Anaplasma phagocytophilum* was demonstrated by polymerase chain reaction and seroconversion. Tickborne encephalitis virus infection was established by serum immunoglobulin (Ig) M and IgG antibodies.

Ticks transmit several bacteria, viruses, and parasites capable of infecting and causing diseases in humans. In general, only a small proportion of bites from infected ticks result in infection, and only a fraction of these infections result in clinical illnesses such as Lyme borreliosis, ehrlichioses, rickettsioses, tickborne encephalitis (TBE), and babesiosis. Infection does not inevitably indicate the presence of the illness nor does it always allow for a reliable explanation of all the signs and symptoms a patient may have. Many distinct causative agents transmitted by the same vector make coinfection and, consequently, the simultaneous presence of more than 1 tickborne disease possible. Human coinfection may occur from a bite of a single tick that transmits multiple pathogens or from multiple tick bites; sequential infections can occur from bites taking place at different times.

Although several reports of coinfection (some of them most probably sequential infections) with tick-transmitted agents have been made, rather limited information exists on the simultaneous clinical features of corresponding illnesses (1). Reports from the United States involve cases with concurrent Lyme disease, babesiosis, or human granulocytic ehrlichiosis (HGE). In addition, U.S. studies indicate that the frequency of simultaneous diseases caused by infection with >1 tickborne pathogen is usually low and varies among geographic locations (1,2). Data from Europe are limited to the reports on coinfections with TBE virus and *Borrelia burgdorferi* sensu lato (s.l.) in patients with acute meningitis (3–7). Other combinations that could have also been possible in European populations, including infections with *Anaplasma phagocytophilum*, *B. burgdorferi* s.l., or TBE virus, have been so far indicated only by the findings of serosurvey studies (8–11).

Slovenia is a small central European country where TBE, Lyme borreliosis, and HGE are known to be endemic. Residents of Slovenia are often exposed to ticks and thus at risk of acquiring infection with multiple tickborne pathogens (7). We report on a patient with TBE and HGE in whom infection with *A. phagocytophilum* was demonstrated by polymerase chain reaction (PCR) and seroconversion, and TBE virus infection was established by the presence of immunoglobin (Ig)M and IgG antibodies to TBE virus in serum.

## Case Report

On May 2, 2003, a 47-year-old-woman, who lived in northwestern Slovenia, was admitted to the Department of Infectious Diseases, University Medical Centre Ljubljana, Slovenia, with a 7-day history of fever <40.0°C, severe headache, nausea, dry cough, malaise, intense myalgia, and arthralgia. She recalled having sustained 3 tick bites on her abdomen during the previous month. The last bite occurred 14 days before onset of her illness while she was walking in the woods near her home; no skin lesions appeared at the site of the bites. Her previous medical history was unremarkable. She had not been vaccinated against TBE, nor had she traveled outside Slovenia during the last few years.

When she sought treatment, her body temperature was 38.5°C, pulse rate was 90 beats/min, and blood pressure was 110/70 mm Hg. With the exception of fever, the physical examination did not show any notable abnormality; rash and meningeal signs were not present. Routine laboratory tests showed leukopenia, thrombocytopenia, abnormal liver function test results, elevated concentration of serum C-reactive protein, and elevated procalcitonin levels (Table 1). A chest radiograph showed no abnormalities. Amoxicillin-clavulanic acid was prescribed. The fever subsided in 3 days (on day 10 after the onset of her illness), and the patient was discharged from the hospital. Her condition improved, but the headache persisted and intensified. On May 13, she was reexamined in our department, and lumbar puncture was performed. Cerebrospinal fluid examination showed normal protein and glucose concentrations but a mildly elevated number of leukocytes (7 x 10^6^/L). At subsequent evaluations, the patient reported feeling better. On day 20 after onset, the control laboratory test results, including liver function test results, were within the normal range.

**Table 1:** Hematologic and blood chemistry values

Variable	Day of illness		
	8	9	12
Leukocytes (normal, 4,000–10,000/mm^3^)	1,900	1,800	4,800
Platelets (normal, 150,000–350,000/mm^3^)	22,000	16,000	43,000
Aspartate aminotransferase (normal, 0–36 U/L)	NT*	49	75
Alanine aminotransferase (normal, 0–42 U/L)	NT	72	108
Lactate dehydrogenase (normal, 140–290 U/L)	NT	499	354
C-reactive protein (normal, <5 mg/L)	277	230	61
Procalcitonin level (normal, <0.50 µg/L)	NT	2.6	0.13

*NT, not tested.

Several microbiologic procedures, including those for determining infections with *A. phagocytophilum*, *Ehrlichia chaffeensis*, *B. burgdorferi* s.l., and TBE virus, were performed to elucidate the cause of the illness. Giemsa-stained peripheral blood smear examination by light microscopy for the presence of ehrlichial morulae within leukocytes was negative. Serum samples were tested by an indirect immunofluorescence assay for the presence of specific IgG antibodies to *A. phagocytophilum* (strain USG3 propagated in HL60 promyelocyte cells), IgM and IgG antibodies to *E. chaffeensis* antigens (MRL Diagnostics, Cypress, California, USA), and IgM and IgG antibodies to *B. burgdorferi* s.l (whole cells of a local isolate of *B. afzelii* were used as an antigen). The presence of serum TBE virus IgM and IgG antibodies was assessed by enzyme-linked immunosorbent assay (ELISA) (Dade Behring Marburg GmbH, Marburg, Germany). The results of serologic tests indicating recent infection with *A. phagocytophilum* and TBE virus are depicted in Table 2. Primers Ehr521 and Ehr790, which amplified the 16S rRNA gene of *A. phagocytophilum*, produced a fragment of the expected size (293 bp) in the acute-phase blood specimen (Figure) (12). No nucleic acids were amplified with primers specific for *E. chaffeensis* HE1 and HE3 (Table 2) (13).

**Table 2:** Results of PCR analysis and serum antibody titers to different tickborne pathogens tested at different times after the onset of illness*

Days after onset of illness	PCR† 16S RNA gene		ELISA IgM/IgG‡ TBEV	IFA IgG *A. phagocytophilum*	IFA IgM/IgG *E. chaffeensis*
	*Anaplasma phagocytophilum*	*Ehrlichia chaffeensis*			
12	Pos	Neg	0.614/129	Neg	Neg/neg
14	Neg	Neg	1.369/251	512	Neg/neg
20	–	–	1.187/382	2,048	Neg/neg
45	–	–	0.419/220	512	Neg/neg
144	–	–	0.400/179	512	Neg/neg

*PCR, polymerase chain reaction; ELISA, enzyme-linked immunosorbent assay; IFA, indirect immunofluorescence assay; Ig, immunoglobulin; Neg, negative; Pos, positive; TBEV, tickborne encephalitis virus. †Primers Ehr521 and Ehr790 specific to *A. phagocytophilum* and primers HE1 and HE3 specific to *E. chaffeensis*. ‡Cut-off value for TBEV IgM is 0.272–0.372 and for IgG is 7.5 U/mL, according to the manufacturer's procedure

**Figure:**
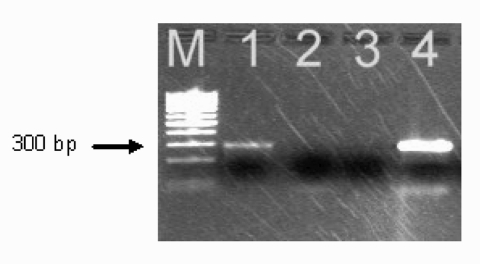
Polymerase chain reaction amplification of *Anaplasma phagocytophilum* DNA from the patient's acute-phase blood sample. Amplified DNA was separated by electrophoresis through the 2% agarose gel stained with ethidium bromide. Lane 1, patient sample (note the presence of the band at ≈293 bp); lane 2, negative sample; lane 3, negative control (no-DNA template control); lane 4 positive control (DNA extracted from the cultured isolate of *A. phagocytophilum*). Lane M represents a 100-bp DNA ladder for estimation of molecular sizes.

## Conclusions

Few reports have been published on serologic evidence of coinfection with TBE virus and *A. phagocytophilum* in Europe. The results on groups of persons representing different risk categories for tick exposure in Switzerland provided serologic evidence of coinfection with *A. phagocytophilum* and TBE virus (9). Weber et al., who retrospectively tested serum specimens of patients with Lyme borreliosis or TBE for antibodies to *A. phagocytophilum*, reported similar findings (10). Although serologic data suggested coinfection with *A. phagocytophilum* and TBE virus, no confirmation of acute HGE was obtained among the residents of Switzerland. In the Czech Republic, where TBE is endemic, among 67 patients hospitalized for TBE, 6 (9%) were seropositive to *A. phagocytophilum* (11). In Slovenia, the background seroprevalence of HGE in children and young adults (15%) was found to be similar to that of Lyme borreliosis (15%) and TBE (13%) (14). In addition, a prospective study was performed to establish the etiologic agents of acute febrile illnesses that occurred within 6 weeks after a tick bite in residents of Slovenia, by using a combination of microbiologic and clinical criteria. Out of 130 adult patients, 36 (28%) had laboratory evidence of TBE virus infection (all had clinically confirmed disease), whereas 4 of 22 (17%) patients with the evidence of *A. phagocytophilum* infection, had confirmed HGE. Infection by multiple organisms (>1) was found in 19 (15%) of 130 patients. Four of them had confirmed TBE and also met the study criteria for probable HGE but not for confirmed HGE (7).

The clinical signs and symptoms of HGE are unspecific and usually consist of fever, headache, chills, malaise, myalgia, or arthralgia, which often occur after a tick bite. Laboratory analysis shows leukopenia, thrombocytopenia, lymphopenia, elevated activity of hepatic enzymes, and an elevated concentration of C-reactive protein (7). However, leukopenia and thrombocytopenia are common not only in HGE patients but also during the initial phase of TBE, in which they were found in 67% and 71% of patients, respectively (15). For most patients in whom TBE develops, the disease has a biphasic course. Our patient, however, had a monophasic course of febrile illness, which was observed in 27% of patients with TBE in Slovenia (16).

In our report, the diagnosis of TBE was established by the presence of a febrile illness after a tick bite with headache, mild lymphocytic pleocytosis, and demonstration of serum IgM and IgG antibodies to TBE virus by ELISA, the serologic method of choice with the specificity 99.9% for IgM and 99.5% for IgG and the sensitivity 99.8% and 96.8% for IgM and IgG, respectively (17). The presence of TBE IgM antibodies 144 days after the onset of illness in our patient (Table 2) is not surprising because the IgM antibodies as a rule persist for several months after acute infection (17). However, changes in antibody titers as demonstrated in our patient attest for recent infection. The febrile illness associated with leukopenia, thrombocytopenia, an elevated concentration of serum C-reactive protein, and the demonstration of *A. phagocytophilum* infection by seroconversion as well as a positive PCR result also met the criteria for confirmed HGE. To our knowledge, the patient reported herein represents the first case of concurrent confirmed TBE and confirmed HGE.

Some reports suggest that coinfection with more than 1 tickborne pathogen may influence the natural history of each of the corresponding diseases, making the clinical course more severe and the outcome less favorable (1). In spite of proven coinfection and fulfillment of criteria for confirmed TBE and HGE (and the absence of treatment with doxycycline, which could have influenced the natural course of HGE), our patient had a relatively mild illness with uneventful recovery. However, the information obtained from a single patient does not allow for a reliable conclusion on the potential influence of TBE virus and *A. phagocytophilum* coinfection on the clinical features and course of the combined illness.
